# Increased Expression of Pregnancy Up-Regulated Non-Ubiquitous Calmodulin Kinase Is Associated with Poor Prognosis in Clear Cell Renal Cell Carcinoma

**DOI:** 10.1371/journal.pone.0059936

**Published:** 2013-04-25

**Authors:** Song Wu, Zhaojie Lv, Yong Wang, Liang Sun, Zhimao Jiang, Congjie Xu, Jun Zhao, Xiaojuan Sun, Xianxin Li, Lijun Hu, Aifa Tang, Yaoting Gui, Fangjian Zhou, Zhiming Cai, Rongfu Wang

**Affiliations:** 1 Institute of Immunology, Zhongshan School of Medicine, Sun Yat-sen University, Guangzhou, Guangdong, China; 2 Shenzhen Second People’s Hospital, The First Affiliated Hospital of Shenzhen University, Shenzhen, Guangdong, China; 3 First Clinical Medical College, Anhui Medical University, Hefei, Anhui, China; 4 Institute of Urology, Shenzhen PKU-HKUST Medical Center, Shenzhen, Guangdong, China; 5 Department of Urology, Sun Yat-Sen University Cancer Center, Guangzhou, Guangdong, China; Johns Hopkins University, United States of America

## Abstract

**Purpose:**

The aims of this study were to evaluate the clinical significance and potential prognostic value of pregnancy up-regulated non-ubiquitous calmodulin kinase (PNCK) in clear cell renal cell carcinoma (ccRCC) patients.

**Materials and Methods:**

The expression of *PNCK* mRNA was determined in 24 paired samples of ccRCCs and adjacent normal tissues using real-time RT-PCR. The expression of PNCK was determined in 248 samples of ccRCCs and 92 paired samples of adjacent normal tissues by immunohistochemical analysis. Statistical analysis was performed to define the relationship between PNCK expression and the clinical features of ccRCC.

**Results:**

The mRNA level of *PNCK* was significantly higher in tumorous tissues than in the adjacent non-tumorous tissues (p<0.001). An immunohistochemical analysis of 92 paired tissue specimens showed that PNCK expression was higher in tumorous tissues than in the adjacent non-tumorous tissues (p<0.001). Moreover, there was a significant correlation between the PNCK expression and various clinicopathological parameters such as Fuhrman grade (p = 0.011), tumor size (p<0.001), T stage (p<0.001) and N stage (p = 0.015). Patients with higher PNCK expression had shorter overall survival time than those with lower PNCK expression (p<0.001). Multivariate analysis indicated that PNCK expression was an independent predictor for poor survival of ccRCC patients.

**Conclusions:**

To our knowledge, this is the first study that determines the relationship between *PNCK* and prognosis in ccRCC. We found that increased PNCK expression is associated with poor prognosis in ccRCC. *PNCK* may represent a novel prognostic marker for ccRCC.

## Introduction

Renal cell carcinoma (RCC) accounts for 3–4% of all human malignancies and it is the 10th leading cause of cancer related death in men. [1] RCC comprises a heterogeneous group of epithelial neoplasms with diverse biologic potential and variable clinical outcomes. [2] ccRCC is the most common type of RCC that occurs in adults and associated with worse prognosis compared with other two major subtypes of chromophobe and papillary RCC. [3], [4] And the 5-year disease-specific survival rate is 50–69%, compared with 67–87% for papillary RCC and 78–87% for chromophobe RCC. [5], [6].

The radical or partial nephrectomy remains the mainstay of curative treatment. However, in most cases, RCC is resistant to chemotherapy and radiotherapy. In addition, the response rate to commonly used immunotherapy with interleukin-2 or interferon-alpha is limited to 10%–20% and mostly the response is partial. To date, targeted therapy of tyrosine kinase inhibitors are widely used as first- and second-line treatments in advanced RCC. However, most of the treatments are not curative and side effects associated with targeted therapy can not be ignored.[7]–[12] Thus, new correlative markers and therapeutic agents are always awaited.

In the previous study, we did a massively parallel sequencing analysis in the 10 ccRCC patients which showed that PNCK was the most significantly upregulated gene in ccRCC in comparison to normal tissues. [13].

*PNCK* is a unique member of the calmodulin kinase I family. It is the most homologous to calmodulin kinase I within the catalytic domain and predominantly expressed in the central nervous system. [14], [15] On the basis of studies, *PNCK* is found to play a role in cytoplasmic and nuclear signal transduction. [16], [17] However, the detailed molecular and genetic understanding of how *PNCK* contributes to the malignant ccRCC remains largely unknown. The possible association between *PNCK* and the development or progression of ccRCC has not been clarified.

Thus, we aimed at exploring the expression of *PNCK* and its clinical significance, which might offer new promise for the clinical evaluation as well as future tumor-specific anticancer approaches.

## Results

### Real-time RT-PCR Analysis of *PNCK* Expression

**Figure 1:**
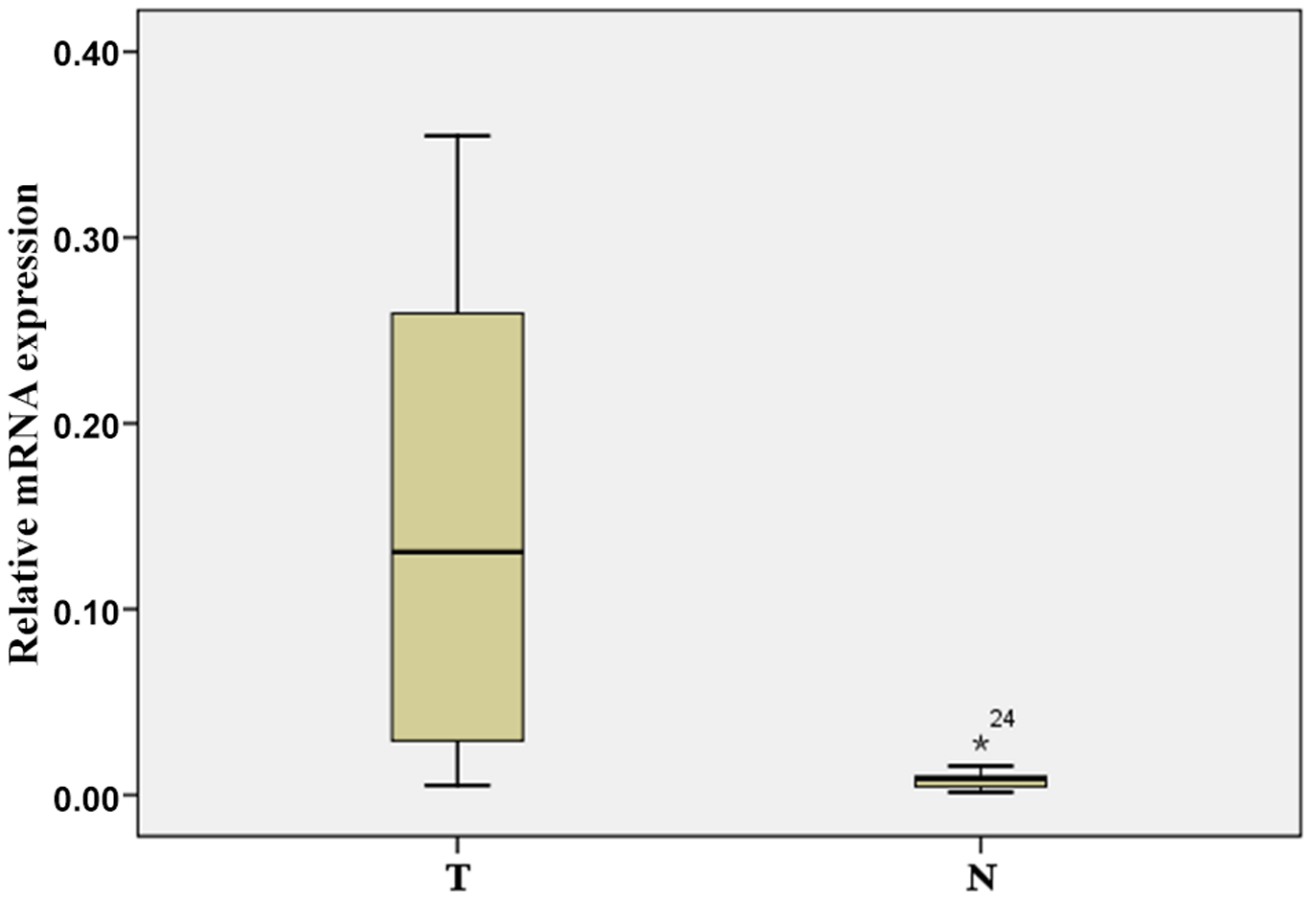
Real-time quantitative RT-PCR analysis of PNCK expression. The relative expression of PNCK mRNA in ccRCC tumor tissue samples was higher than that in the paired adjacent normal (N) tissue samples (n = 24, P<0.001). The bottom and the top of the box represent the 25th and the 75th percentile, respectively, and the band near the middle of the box is the 50th percentile (the median). The ends of the whiskers represent the 2.5th percentile and the 97.5th percentile.

The transcription level of *PNCK* was determined by real-time RT-PCR assays of 24 ccRCC tumor samples and the paired adjacent normal tissue samples. In 24 tumor samples, the mRNA level of *PNCK* was significantly higher than that in the adjacent normal tissue sample (p<0.001, paired-sample t tests, Figure 1).

### Immunohistochemical Analysis of the Expression of PNCK Protein in 92 Paraffin-embedded ccRCC Samples (T) and the Paired Adjacent Normal Renal Tissue (N)

**Figure 2:**
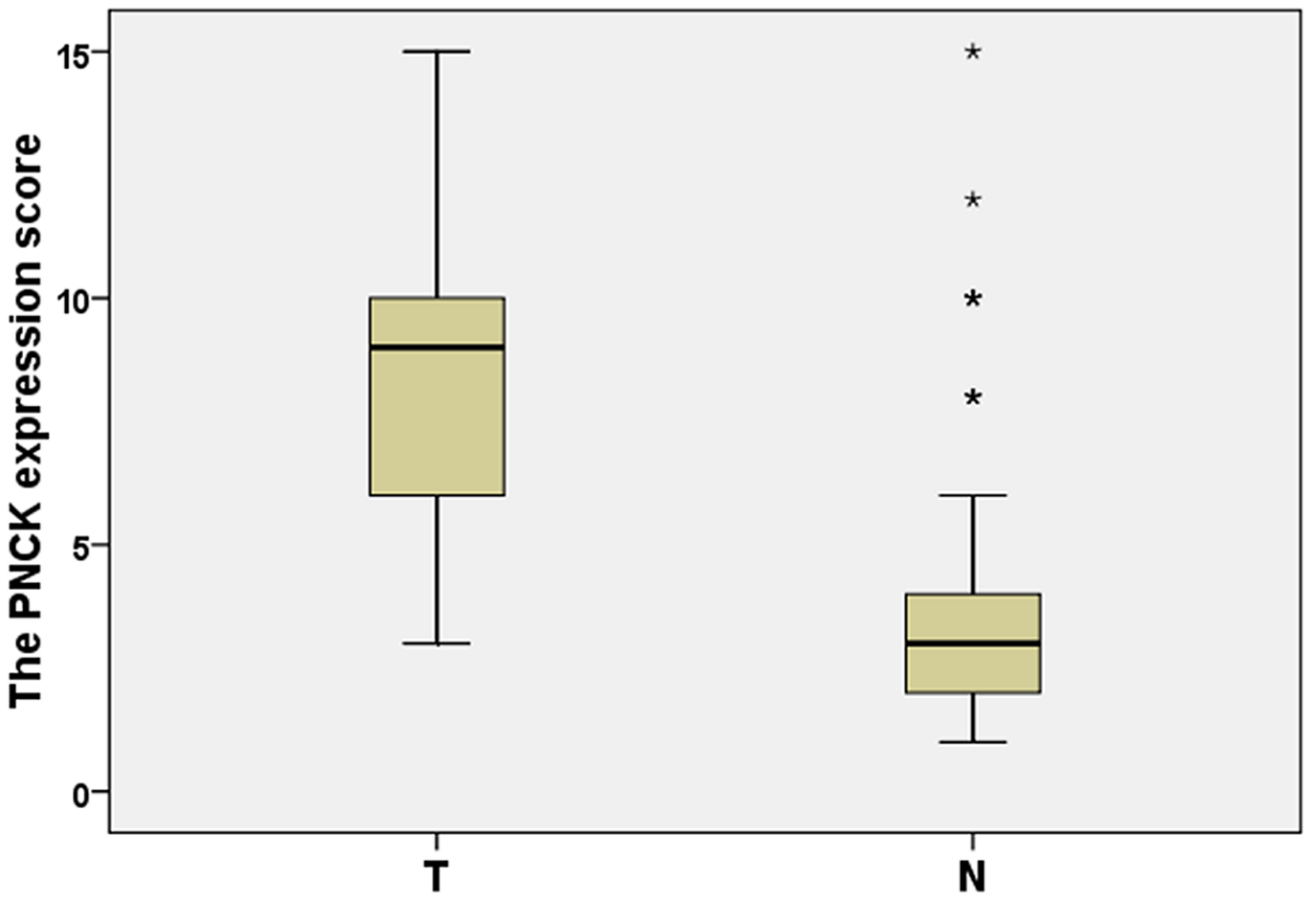
Increased protein expression of PNCK in ccRCC. The relative protein expression of PNCK in ccRCC tumor (T) tissue samples was higher than that in the paired adjacent normal (N) tissue samples (n = 76, P<0.001). The bottom and top of the box are the lower and upper quartiles, and the band near the middle of the box is the median. The ends of the whiskers represent the 2.5th percentile and the 97.5th percentile. Four black stars represent the special value outliers.

**Figure 3:**
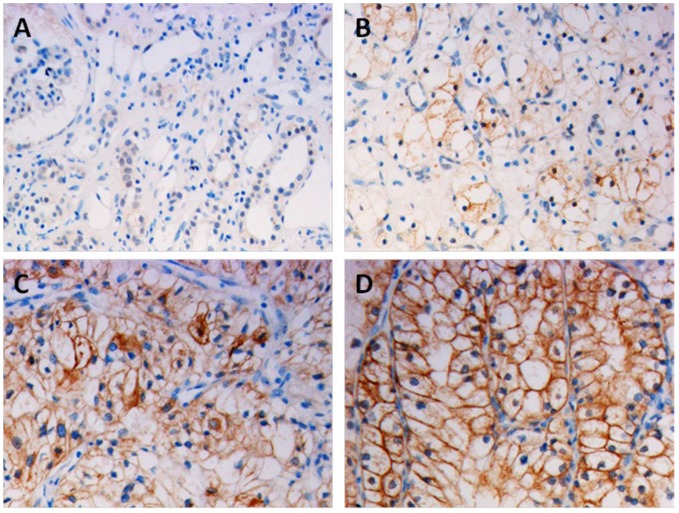
Immunohistochemical analysis of the expression of PNCK protein. PNCK is mainly localized within the nuclei and cytoplasmic. Immunostaining of the adjacent normal tissue samples(A) and the ccRCC tumor tissue samples(B) showed a sharp contrast between the negatively stained infiltrative tumorous area. (B): Negative or weak PNCK staining in cancerous tissue (400×). (C): Moderate PNCK staining in cancerous tissue(400×). (D): Strong PNCK staining in most of tumor cells (400×).

Expression and subcellular localization of protein were determined by immunohistochemical analysis in 248 paraffin-embedded ccRCC tissues and 92 paired specimens of adjacent normal tissues (Figure 2). In normal renal tissue, specific PNCK was localized mainly in the cytoplasm of renal cells in the form of yellow-brown granules (Figure 3A). The PNCK protein expression in 76 tumor tissue samples was higher than that in the adjacent normal tissue samples (p<0.001, paired-sample *t* test, Figure 3B).

Besides, immunohistochemical analysis showed that in the same 24 paired tissue specimens tested for mRNA level, the PNCK protein expression was higher in tumorous tissues than in the adjacent non-tumorous tissues (p<0.001).

### Immunohistochemical Analysis of PNCK Expression in 248 ccRCC Samples (T) and its Relationship with the Clinical Feature

To further investigate the effect and the prognostic value of *PNCK*, immunohistochemical analysis was performed to assess the expression of PNCK in 248 ccRCC tissue blocks. Overall, 145 of the 248 tumor samples showed high expression of PNCK (score ≥5), whereas 103 samples showed low expression (score ≤4). The association between the expression of PNCK and various clinicopathological parameters are listed in Table 1. Intense expression of PNCK in ccRCC samples was correlated with Fuhrman grade (p = 0.011), tumor size (p<0.001), T stage (p<0.001) and N classification (p = 0.015), but it was not correlated with gender (p = 0.164), age (p = 0.229), metastasis (p = 0.115) and recurrence (p = 0.985). High expression of PNCK was noted in 48.4%, 87.2%, and 68.8% of T1, T2, and T3/4 stage of ccRCCs (p<0.001, χ2 test). High expression of PNCK was observed in 48.7% and 76.7% of ccRCCs with size ≤7 cm and >7 cm respectively (p<0.001, χ2 test). High expression of PNCK was seen in 55.9% and 80.8% of N0 and N1/2 stage ccRCCs respectively (p = 0.015, χ2 test).

**Table 1:** Correlation between PNCK expression and clinical pathologic features of the patients with clear cell renal cell carcinoma.

Clinical-pathologic variables	n	PNCK expression		?2	p		
		Low	High				
All cases	248	103	145	1.938		0.164	
Male	164	63	101				
Female	84	40	44				
Age (yrs)				1.449			0.229
>50	122	46	76				
≤50	126	57	69				
Fuhrman Grade				11.119 0.011			
I	119	62	57				
II	71	24	47				
III	41	13	28				
IV	17	4	13				
Tumor size (cm)				18.110			<0.001
≤7	162	83	79				
>7	86	20	66				
T stage				26.497			<0.001
T1	161	83	78				
T2	39	5	34				
T3,	41	11	30				
T4	7	4	3				
N stage				5.949			0.015
N0	222	98	124				
N+	26	5	21				
Metastasis				2.491			0.115
No	225	97	128				
Yes	23	6	17				
Recurrence				0.001			0.989
No	224	93	131				
Yes	24	10	14				

### Survival Analysis

**Figure 4:**
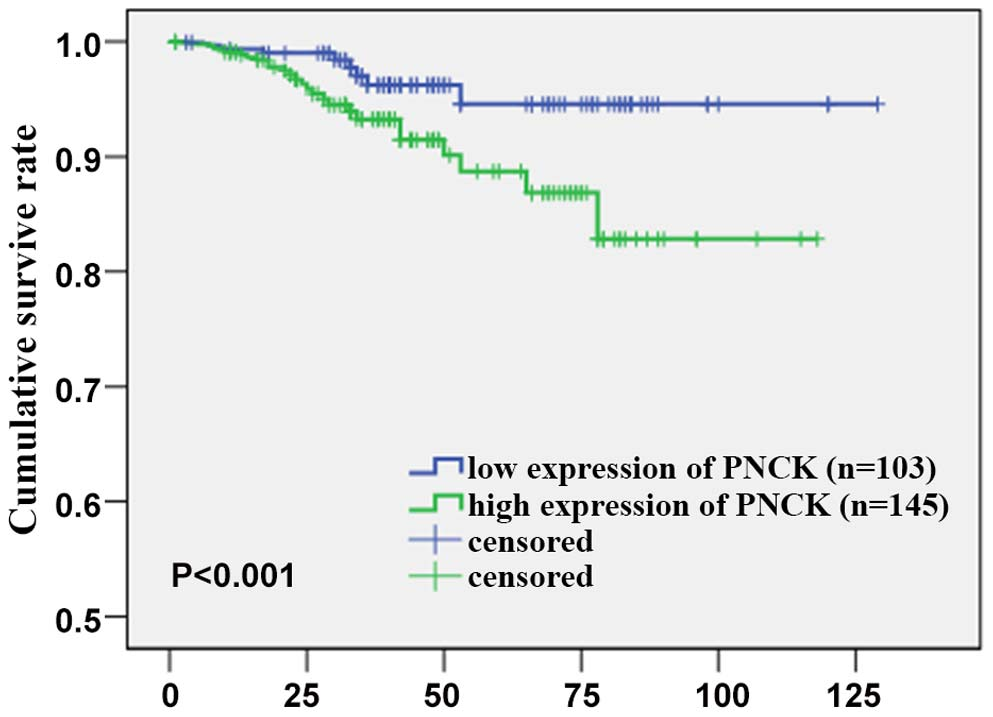
Overall survival. Kaplan-Meier survival analysis of primary ccRCC patients (n = 248) after surgical resection with low PNCK expression (n = 103) and high PNCK expression (n = 145). The survival rate for patients in the PNCK high group was significantly lower than that for patients in the PNCK low group (log-rank test, p<0.001).

Kaplan-Meier analysis and the log-rank test were used to calculate the effect of the PNCK expression on survival. The 5-year survival in the group of patients with low PNCK expression was 95%, but it was 88% in the group of patients with high PNCK expression (Figure 4). The log-rank test showed that survival rates were significantly different between these 2 groups (p<0.001). Univariate Cox regression analysis showed that tumor size, T stage, N stage, metastasis, Fuhrman grade and PNCK expression were significantly associated with overall survival (Table 2). Furthermore, multivariate Cox regression analysis revealed that PNCK expression(p<0.001 ), N stage(p<0.001 ) and Fuhrman grade(p<0.001 ) were independent predictors for the overall survival of ccRCC patients (p<0.001, p<0.001, p<0.001; Table 2), whereas the other factors were not independently related to the survival of ccRCC patients.

**Table 2:** Cox Regression analysis of the overall survival rates associated with different prognostic variables in patients with ccRCC.

Variables	Univariate analysis		Multivariate analysis	
	Hazard ratios (95% confidence interval)	p	Hazard ratios (95% confidence interval)	p
Tumor size	(5.447–7.102)	<0.001		
T stage	(2.397–2.692 )	<0.001		
N stage	(3.644–4.625 )	<0.001	(2.975–2.113)	<0.001
Metastasis	(6.658–8.971 )	<0.001	(0.100–6.355)	0.747
Fuhrman grade	(2.280–2.557)	<0.001	(2.379–2.754)	<0.001
PNCK	(2.417–3.239 )	<0.001	(0.633–0.700)	<0.001

## Discussion

Epidemiological studies have shown that rising kidney cancer incidence has been reported in most countries over the past three decades. In the United States, incidence rate of renal cell cancer, the predominant subtype of kidney cancer, rose through the mid-2000 s. RCC is the eighth most commonly diagnosed malignancy and the tenth leading cause of cancer deaths among men in the United States. [18], [19] ccRCC is a renal cortical tumor typically characterized by malignant epithelial cells with clear cytoplasm and a compact-alveolar growth pattern. Clinically, treatment options include surgery, chemotherapy, radiation therapy, immunotherapy, or a combination of these approaches. Unfortunately, ccRCC is resistant to radiation therapy and chemotherapy. Immunotherapy (or cytokine therapy) with interleukin-2 (IL-2) and interferon-α (IFN-α) which provided modest survival benefit, has a more favorable toxicity profile.

In recent years, an improved understanding of the molecular basis of RCC has led to the development of RCC therapy. Currently, four VEGF receptor inhibitors (sorafenib, sunitinib, pazopanib and axitinib), one anti-VEGF monoclonal antibody (bevacizumab) and two inhibitors of the mammalian target of rapamycin (mTOR) pathway (temsirolimus and everolimus) have been approval by the Food and Drug Administration (FDA) for the treatment of advanced RCC. Studies also showed that carbonic anhydrase IX (CAIX) gene and von Hippel–Lindau (VHL) gene can stratify patients with clear cell RCC into distinct groups with regards to clinicopathological variables and prognosis. [20].

However, not all the patients treated with these targeted therapies have a substantial clinical benefit and none of those therapies have been proven to improve overall survival. Virtually, all patients exhibit disease progression at a median of 5–11 months. Moreover, some critical clinical problems are still there to be solved such as chronic toxicity, adverse drug reactions as well as high costs. The molecular mechanisms of the initiation and progression of ccRCC remain unclear. Therefore, it is still important to search for more effective correlative markers and therapeutic agents. [21], [22].

One therapeutic target that has been drawing attention lately is *PNCK*, which was recognized as one of the most highly over-expressed gene in human ccRCC by methods of massive parallel sequencing analysis in 10 ccRCC patients in our previous study. [13].

*PNCK*, a novel CaM kinase, is up-regulated in the mouse mammary gland during pregnancy in a subset of epithelial cells, suggesting that PNCK expression is correlated with differentiation and apoptosis. [23] *PNCK* was previously found to be over-expressed in the transformation of human breast cancer. It indicated that *PNCK* may function in signal transduction cascades involved in mammary development and carcinogenesis. [24] A recent report also revealed that *PNCK* mediates the protea-lysosomal degradation of EGFR protein. [25] *PNCK* induces ligand-independent EGFR degradation most likely through perturbation of Hsp90 chaperone activity and EGFR degradation is coupled to proteasomal degradation of *PNCK*. Thus, *PNCK* may represent a promising target for therapeutic intervention in EGFR-regulated oncogenesis. [26] The chromosomal localization of *PNCK*, along with its tissue-specific and restricted pattern of spatial expression during development, indicate that *PNCK* may also be involved in a variety of developmental processes. [27].

Here, to our knowledge, the key feature of our study is that it is the first time to report the clinical significance of *PNCK* in ccRCCs. This is also the first study aimed at evaluating the possibility of using *PNCK* as a clinically potential indicator for disease progression, as well as a prognostic marker for patient survival in tumors.

In our previous study, immunohistochemical analysis showed that PNCK expression was moderate to high in ccRCCs, while it was low in the adjacent normal tissues. [11] Here, we found that PNCK expression was increased in a large number of human clinical ccRCC samples. The increased expression of PNCK was correlated with Fuhrman grade, tumor size, T stage and N stage. While high PNCK expression is not correlated with metastasis and recurrence condition. This may be related to the relative short average follow-up. Besides, some unmeasured differences may exist and distort the study results.

Furthermore, in the survival analysis, we found that patients with lower PNCK expression had longer survival time, and those with higher PNCK expression had shorter survival time. In the univariate Cox regression analysis, tumor size, T stage, N stage, metastasis, Fuhrman grade and PNCK expression were significantly associated with overall survival. In addition, multivariate Cox regression analysis revealed that PNCK expression, Fuhrman grade and N stage were independent predictors for the overall survival of ccRCC patients.

Clinically, the prediction of prognosis in patients with ccRCC is based on clinical and pathologic factors such as the patients’ age, overall health the TNM stage and pathologic stage of the cancer. Our study showed that high-expression of PNCK indicated poor prognosis for patients with ccRCC, consistent with previous reports of breast cancer. [21] Thus, *PNCK* could be a valuable prognostic marker for ccRCC patients and an additional increase in predictive accuracy will be likely to achieved. [28], [29].

To the best of our knowledge, this is the first study to report the clinical significance and the possibility of using *PNCK* as therapeutic target gene in ccRCC. However, detailed molecular mechanisms need to be investigated further. In addition, some current studies have suggested that loss of the gene 3p/*VHL* are associated with improved survival, and that loss of 4 p, 14 q, 9 p and Y are all further important predictors of prognosis. [30] Thereby, more studies are required to explore the relationship between the *PNCK* gene and other abovementioned molecular markers.

It has to be pointed out that our study was a single hospital-based and retrospective study. In order to further demonstrate our study, a multicenter or community-based prospective study with more extensive collection of potential cofounders is also required.

### Conclusion

In summary, we demonstrated the up-regulation of *PNCK* in ccRCC and its correlation with poor prognosis by using a large number of clinical samples. Our results indicate that measurement of *PNCK* expression in primary ccRCC can help stratify the patients for prognosis. As a potential tumor suppressor in primary ccRCC, *PNCK* is potentially an excellent target from a therapeutic and prognostic standpoint. However, further validation study is needed before PNCK gene becoming a prognostic marker and being integrated into prognostic models.

## Materials and Methods

### Patients and Tissue Specimens

For real-time RT-PCR analysis, a total of 24 tissue samples of ccRCCs and 24 corresponding adjacent normal tissues from patients were studied. The 24 patients comprised 14 male (58.3%) and 10 female (41.7%) patients, with a median age of 51.0 years (range: 38–75 years). All the samples were obtained by radical nephrectomy at the Cancer Center of Sun Yat-sen University between January 2009 and June 2010. None of them received preoperative treatment such as radiation and chemotherapy. Radical nephrectomy specimens were immersed in RNA later (Qiagen; Germany) immediately after surgery, stored at 4°C overnight to allow thorough penetration of the tissue, and then frozen at −80°C until examination. In addition, we collected 248 paraffin-embedded samples of ccRCCs and 92 adjacent normal renal tissue samples (between 1999 through 2007) for the immunohistochemical analysis. The characteristics of these 248 patients are listed in Table 1. The histological and clinical diagnosis of the tumors in all these patients was performed by the Cancer Center of Sun Yat-sen University. All patients enrolled in the study have given written informed consent. This study was approved by the institutional review board of Sun Yat-sen University. The disease stage of each patient was classified or reclassified according to the 2002 American Joint Committee on Cancer (AJCC) staging system. [31].

### Real-time RT-PCR

Total RNA was isolated using the TRIzol solution (Invitrogen; Carlsbad, CA) according to the manufacturer’s instructions. The first-strand cDNA, synthesized from 2 µg of total RNA using M-MLV reverse transcriptase (Fermentas; American), was then subjected to real-time quantitative PCR after the DNA contamination being removed by RNase-free DNase. The primer pairs used for RT-PCR amplification of the relative mRNA of *PNCK* and *GAPDH* (as an internal control) were as follows: *PNCK* sense strand: 5′-TATGCCACGCCCTTTGAG-3′, *PNCK* antisense strand: 5′-CACAGCAGGATGTAGGAGATGA-3′, *GAPDH* sense strand: 5′-GCTCTCTGCTCCTCCTGTTC-3′, *GAPDH* antisense strand: 5′-GACTCCGACCTTCACCTTCC-3′. Applied Biosystems (ABI 7000) real-time PCR machine was used for Gene-specific amplification, with a 20-µl PCR reaction mixture containing 1 µl of cDNA (synthesized as described above), 10-µl SYBR Green master mix (Invitrogen; Carlsbad, CA), and 40 nM of each pair of oligonucleotide primers. They were mixed and amplified with the following regimen: 1 cycle of 50°C (2 min) and 95°C (2 min) and 40 cycles of denaturation at 95°C (15 sec); annealing at 55°C (30 sec); extension at 72°C (40 sec). Regression curves were calculated for each sample, and the relative amount of mRNA was calculated from the threshold cycles by using the software provided with the instrument (Version 17.0 SPSS Inc.). The expression of *GAPDH* (the internal control gene), was used to normalize for variance. Relative expression levels of the target genes were normalized to the geometric mean of the internal control gene, *GAPDH*. The data was analyzed using the comparative threshold cycle (2^−ΔCT^) method.

### Immunohistochemical Assay

In order to examine the PNCK expression, the 248 samples of ccRCC and 92 samples of adjacent normal renal tissues were used to perform immuhistochemical reactions, according to the following classical protocols. Formalin-fixed, paraffin-embedded tissues were sectioned at 5-µm and baked at 65°C for 30 min. All sections were dewaxed in xylene and rehydrated through a sequence of decreasing concentration of alcoholic solutions; endogenous peroxydase activity was quenched by 3% hydrogen peroxide in methanol followed by incubation for 30 min at room temperature. Sections were microwave-pretreated in 0.01 M citrate buffer (pH 6.0) for antigen retrieval. Nonspecific binding was blocked by incubation with bovine serum albumin. The PNCK protein was detected by using a mouse monoclonal antibody against PNCK (Sigma). The specimens were incubated overnight at 4°C with anti-PNCK antibody (1∶250). The negative control for immunohistochemical analysis was obtained by replacing the primary antibodies with an antibody diluent. After being washed in phosphate buffered saline (PBS), the sections were treated with MaxVision™ HRP-Polymer anti-Mouse IHC Kit (Maixin Bio; Fujian, China) at 37°C for 15–20 min. The tissue sections were immersed in 3-amino-9-ethyl carbazole, counterstained with Mayer’s hematoxylin, dehydrated, and finally mounted in Crystal Mount.

The formalin-fixed, paraffin-embedded sections were reviewed for the degree of immunostaining and scored by 2 independent observers. All cases’ IHC scoring has been confirmed and agreed on by the two independent pathologists. If there are disagreements, a third independent observer will be needed to make sure the accuracy of results. The proportion of cells expressing PNCK varied from 0% to 100%, and the intensity of staining varied from weak to strong. The proportion of PNCK- expressing tumor cells was scored as follows: 0, no positive cells; 1, 0%–5%; 2, 6%–25%; 3, 26%–50%; 4, 51%–75%; and 5, >75% according to Tsuchiya et al. The staining intensity was graded according to the mean optical density: 0, no staining; 1, weak staining (light yellow); 2, moderate staining (yellow brown) and 3, strong staining (brown) as shown in figure3.B,C,D. [32], [33] Staining index was calculated as the multiplication of staining intensity score and the proportion of PNCK-positive tumor cells. We evaluated PNCK expression in benign kidney tissue and malignant lesions on the basis of the staining index values, with scores of 0, 1, 2, 3, 4, 5, 6, 8, 9, 10, 12, and 15. The cutoff values for PNCK expression were chosen on the basis of a measure of heterogeneity in overall survival rates, which was calculated using the log-rank test. There was no case with score 5. An optimal cutoff value was identified: a staining index score of ≥5 was considered as high PNCK expression, whereas a staining index score of ≤4 was considered as low PNCK expression.

### Statistical Analysis

All statistical analysis was analyzed using SPSS 17.0 software package. The paired-sample *t* tests were used to analyze the significance of the differences in mRNA and protein expression between ccRCCs and the adjacent normal tissues in the real-time RT-PCR and immunohistochemical assays. The χ2 test for proportion was used to analyze the relationship between PNCK expression and clinical significance. Survival rate curves were drawn according to the Kaplan-Meier method, and differences between the curves were analyzed by applying the log-rank test. The influence of each variable on survival was analyzed by multivariate analysis using the Cox proportional hazards model. Stepwise Cox’s regression analysis was performed to identify the prognostic factors for survival. In all tests, differences with P<0.05 were considered statistically significant.

## References

[1] Jemal A, Siegel R, Ward E, Hao Y, Xu J, et al (2009) Cancer statistics. CA Cancer J Clin 59: 225. 19474385 10.3322/caac.20006

[2] Cheng L, Zhang S, MacLennan GT, Lopez-Beltran A, Montironi R (2009) Molecular and cytogenetic insights into the pathogenesis, classification, differential diagnosis, and prognosis of renal epithelial neoplasms. Hum Pathol 40: 10. 19027455 10.1016/j.humpath.2008.09.009

[3] Ljungberg B, Cowan NC, Hanbury DC, Hora M, Kuczyk MA, et al (2010) European Association of Urology Guideline Group:EAU guidelines on renal cell carcinoma: the update. Eur Urol 58: 398. 20633979 10.1016/j.eururo.2010.06.032

[4] Grignon D J, Che M (2005) Clear cell renal cell carcinoma. Clin Lab Med 25: 305. 15848738 10.1016/j.cll.2005.01.012

[5] Gudbjartsson T, Hardarson S, Petursdottir V, Thoroddsen A, Magnusson J, et al (2005) Histological subtyping and nuclear grading of renal cell carcinoma and their implications for survival: a retrospective nation-wide study of 629 patients. Eur Urol (48(4)) 593–600. 10.1016/j.eururo.2005.04.016 15964127

[6] Cheng L, Zhang S, MacLennan GT, Lopez-Beltran A, Montironi R (2009) Molecular and cytogenetic insights into the pathogenesis, classification, differential diagnosis, and prognosis of renal epithelial neoplasms. Hum Pathol. 40(1): 10–29. 10.1016/j.humpath.2008.09.009 19027455

[7] Wu S, Can W, Lai Y, Ye J (2012) High expression of FER tyrosine kinase predicts poor prognosis in clear cell renal cell carcinoma. ONCOLOGY LETTERS 5: 473–478. 23420638 10.3892/ol.2012.1032 PMC3573111

[8] Mulders PF, Brouwers AH, Hulsbergen-van der Kaa CA, van Lin EN, Osanto S, et al (2008) Renal cell carcinoma (in Dutch; Flemish). Ned Tijdschr Geneeskd 152: 376. 18380384

[9] Woods LS (2010) New therapeutic strategies in renal cell carcinoma.Urol Nurs. 30: 40. 10.7257/1053-816x.2010.30.1.40 20359144

[10] Yang JC, Haworth L, Sherry RM, Hwu P, Schwartzentruber DJ, et al (2003) A randomized trial of bevacizumab, an anti-vascular endothelial growth factor antibody for metastatic renal cancer. N Engl J Med 349: 427. 12890841 10.1056/NEJMoa021491 PMC2275324

[11] Motzer RJ, Hutson TE, Tomczak P, Michaelson D, Ronald M, et al (2007) Sunitinib versus interferon alfa in metastatic renal-cell carcinoma. N Engl J Med 356: 115. 17215529 10.1056/NEJMoa065044

[12] Escudier B, Eisen T, Stadler WM, Szczylik C, Oudard S, et al (2007) Sorafenib in advanced clear-cell renal-cell c arcinoma. N Engl J Med 356: 125. 17215530 10.1056/NEJMoa060655

[13] Zhou L, Chen J, Li Z, Li Xx, Hu Xd, et al (2010) Integrated Profiling of MicroRNAs and mRNAs: MicroRNAs Located on Xq27.3 Associate with Clear Cell Renal Cell Carcinoma. PLoS ONE 5: e15224. 21253009 10.1371/journal.pone.0015224 PMC3013074

[14] Gardner HP, Rajan JV, Ha SI, Copelandb NG, Gilbertb DJ, et al (2000) Cloning, characterization, and chromosomal localization of Pnck, a Ca2/calmodulin-dependent protein kinase. Genomics 63: 279. 10673339 10.1006/geno.1999.6091

[15] Loseth OP, de Lecea L, Calbet M, Danielsonc PE, Gautvik V, et al (2000) Developmental regulation of two isoforms of Ca2+/calmodulin-dependent protein kinase I beta in rat brain. Brain Res 869: 137. 10865068 10.1016/s0006-8993(00)02359-3

[16] Rina S, Jusuf AA, Sakagami H, Kikkawaa S, Kondo H, et al (2001) Distribution of Ca2_/calmodulin-dependent protein kinaseI beta 2 in the central nervous system of the rat. Brain Res 911: 1. 11489438 10.1016/s0006-8993(01)02440-4

[17] Ueda T, Sakagami H, Abe K, Oishi I, Maruo A, et al (1999) Distribution and intracellular localization of a mouse homologue of Ca2/calmodulin-dependent protein kinase Ibeta2 in the nervous system. J Neurochem 73: 2119. 10537072

[18] Chow W-H, Dong LM, Devesa SS (2010) Epidemiology and risk factors for kidney cancer. Nat Rev Urol 7: 245. 20448658 10.1038/nrurol.2010.46 PMC3012455

[19] Wu S, Wang Y, Sun L, Zhang Z, Jiang Z, et al (2011) Decreased expression of dual-specificity phosphatase 9 is associated with poor prognosis in clear cell renal cell carcinoma. BMC Cancer11: 413. 10.1186/1471-2407-11-413 PMC3198720 21943117

[20] Iliopoulos O (2006) Molecular biology of renal cell cancer and the identification of therapeutic targets. J Clin Oncol 24: 5593–5600. 17158545 10.1200/JCO.2006.08.8948

[21] Sabatino M, Kim-Schulze S, Panelli MC, Stroncek D, Wang E, et al (2009) Serum vascular endothelial growth factor and fibronectin predict clinical response to high-dose interleukin-2 therapy. J Clin Oncol (27) 2645. 10.1200/JCO.2008.19.1106 PMC2689845 19364969

[22] Shaheen PE, Bukowski RM (2005) Emerging drugs for renal cell carcinoma. Expert Opin Emerg Drugs (10) 773–795. 10.1517/14728214.10.4.773 16262562

[23] Pugazhenthi S, Nesterova A, Sable C, Heidenreich KA, Boxer LM, et al (2000) Akt/protein kinase B up-regulates Bcl-2 expression through cAMP-response element-binding protein. J Biol Chem. 275: 10761. 10.1074/jbc.275.15.10761 10753867

[24] Gardner HP, Ha SI, Reynolds C (2000) The caM kinase, Pnck, is spatially and temporally regulated during murine mammary gland development and may identify an epithelial cell subtype involved in breast cancer. Cancer research (60) 5571. 11034105

[25] Deb TB, Coticchia CM, Barndt R, Zuo H, Dickson RB, et al (2008) Pregnancy upregulated nonubiquitous calmodulin kinase induces ligand-independent EGFR degradation. Am J Physiol Cell Physiol (295) C365. 10.1152/ajpcell.00449.2007 PMC2518423 18562482

[26] Deb TB, Zuo AH, Wang Y, Barndt RJ, Cheema AK, et al (2011) Pnck induces ligand-independent EGFR degradation by probable perturbation of the Hsp90 chaperone complex. Am J Physiol Cell Physiol 300: C1139. 21325639 10.1152/ajpcell.00167.2010 PMC3093937

[27] Gardner HP, Rajan JV, Ha SI, Copeland NG, Gilbert DJ, et al (2000) Cloning, characterization, and chromosomal localization of Pnck, a Ca(2+)/calmodulin-dependent protein kinase. Genomics 63: 279. 10673339 10.1006/geno.1999.6091

[28] Frank I, Blute ML, Cheville JC, Lohse CM, Weaver AL, et al (2002) An outcome prediction model for patients with clear cell renal cell carcinoma treated with radical nephrectomy based on tumor stage, size, grade and necrosis: The SSIGN score. J Urol. 168: 2395. 10.1016/S0022-5347(05)64153-5 12441925

[29] Sorbellini M, Kattan MW, Snyder ME, Reuter V, Motzer R, et al (2005) A postoperative prognostic nomogram predicting recurrence for patients with conventional clear cell renal cell carcinoma. J Urol. 173: 48. 10.1097/01.ju.0000148261.19532.2c 15592023

[30] Klatte T, Rao PN, de Martino M, LaRochelle J, Shuch B, et al (2009) Cytogenetic Profile Predicts Prognosis of Patients With Clear Cell Renal Cell Carcinoma. J Clin Oncol. 27: 746. 10.1200/JCO.2007.15.8345 19124809

[31] Greene FL, Page DL, Fritz A, Balch CM, Fleming ID, et al (2003) Cancer Staging Manual. Ann Oncol. 14 (2): 345–346.

[32] Wu S, Sun X, Zhu W, Huang Y, Mou L, et al (2012) Evidence for GAL3ST4 mutation as the potential cause of pectus excavatum. Cell Res. 22(12): 1712–1715. 10.1038/cr.2012.149 PMC3515755 23147795

[33] Bao S, Ouyang G, Bai X, Huang Z, Ma C, et al (2004) Periostin potently promotes metastatic growth of colon cancer by augmenting cell survival via the Akt/PKB pathway. Cancer Cell. 5: 329. 10.1016/s1535-6108(04)00081-9 15093540

